# No Differences in Motor Units Discharge Rate Between Females and Males in Explosive Ankle Dorsiflexions

**DOI:** 10.1111/sms.70065

**Published:** 2025-05-11

**Authors:** Anna Grootenhuis, Fiona C. Hiereth, Jakob Škarabot, Marius Oßwald, Alessandro Del Vecchio, Markus Gruber, Luca Ruggiero

**Affiliations:** ^1^ Human Performance Research Centre, Department of Sport Science University of Konstanz Konstanz Germany; ^2^ School of Sport, Exercise and Health Sciences Loughborough University Loughborough UK; ^3^ Neuromuscular Physiology and Neural Interfacing (N‐Squared) Laboratory, Department of Artificial Intelligence in Biomedical Engineering Friedrich‐Alexander‐Universität Erlangen Germany

**Keywords:** ballistic, EMG decomposition, firing rate, motoneurones, rapid, sex differences

## Abstract

Males and females exhibit comparable levels of explosive strength if the rate of torque development (RTD) is considered relative to the maximal voluntary isometric torque (MVT). Given the greater proportion of type I to type II fibers area in muscles of females than males (~20% in tibialis anterior muscle), with slower contractile kinetics, the discharge rate of motor units (MUDR) in explosive efforts may be higher to compensate for the slower muscle contractile properties. Yet, it is to date unknown whether sex differences in MUDR in explosive efforts exist. Twenty‐two females and 12 males (20–34 years) performed electrically evoked and voluntary rapid isometric ankle dorsiflexions. Electrically evoked contractions consisted of three trains of 50 supramaximal stimuli at the common peroneal nerve at 200 Hz. Ten voluntary explosive contractions were then performed while recording high‐density electromyography signals from the tibialis anterior. No significant differences were reported between males and females for both voluntary and electrically evoked relative peak RTD (8.0 ± 2.0 vs. 7.7 ± 0.8 and 6.4 ± 0.7 vs. 6.1 ± 2.1 MVT s^−1^), and for RTD and muscle activity throughout the first 150 ms (*p* > 0.07). Despite no significant differences, these metrics were not equivalent between sexes when assessed with equivalence testing. Time to peak RTD was shorter in males than females for both contraction types (voluntary: 61 ± 12 vs. 74 ± 14 ms; electrically evoked: 24 ± 3 vs. 28 ± 7 ms; *p* < 0.01). MUDR at the beginning of the explosive voluntary efforts was not different between sexes (males: 61 ± 15; females: 67 ± 16 Hz; *p* < 0.23). Overall, despite known sex‐related differences within the skeletal muscle, the control of rapid torque production is not different between sexes.

**Abbreviations**
EMG: electromyography
IMP: impulse
*M*_max_
: maximal compound muscle action potential
MUDR: motor units discharge rate
MVT: maximal voluntary isometric torque
pRTD: peak rate of force or torque development from a 10‐ms moving window
pRTDf: peak rate of force or torque development from the RTD function
RMS: root‐mean‐square
RTD: rate of force or torque or force development

## 1 Introduction

Explosive strength is the fundamental neuromuscular skill of producing the highest amount of force in the minimum time [1, 2]. It is typically quantified as the rate of force or torque development (RTD) of a muscle group during a maximal voluntary effort [3, 4]. Explosive strength is key for functional outcomes in sports and in a multitude of clinical conditions [5, 6, 7]. Consequently, several studies have looked at the underlying mechanisms behind explosive strength production and its conditioning (see review of [2]).

Females and males present significant differences in fundamental neuromuscular capabilities [8]. Males have greater levels of maximal strength and power [9], driven primarily by muscular mechanisms such as a greater cross‐sectional area of all muscle fiber types, particularly of type II fibers [10, 11]. However, females outperform males in the context of neuromuscular fatigability when exercising at the same intensity relative to the maximal voluntary contraction torque (MVT) or power output [12, 13]. One potential difference is the neuromodulation of muscle force or joint torque between sexes. Several studies have focused on the discharge rate of motor units (MUDR) in maximal and submaximal isometric contractions (e.g., [14, 15, 16, 17]; see also review of [18]). Typically, across several lower and upper limb muscles (e.g., tibialis anterior, vastus lateralis and medialis, first dorsal interosseus, biceps brachii), females exhibit higher MUDR than males in submaximal isometric contractions at the same relative intensity, but lower MUDR in maximal efforts [14, 15, 17, 19, 20, 21, 22, 23]. Furthermore, a recent study found larger estimates of persistent inward currents in females from motor units of the tibialis anterior and ankle plantarflexors, suggesting greater neuromodulatory input to the motoneurones relative to males, despite comparable levels of MUDR in submaximal isometric contractions [24].

Given the importance of explosive strength for movement, previous studies have examined sex differences in rapid force production, in elbow flexors [25, 26], knee flexors and extensors [27, 28, 29], and ankle dorsiflexors [30, 31]. The consensus is that when MVT is accounted for, RTD in explosive efforts is not different between males and females [26, 28, 29, 30, 31]. Likewise, muscle activity (measured with surface bipolar electromyography; EMG) does not differ between sexes [28, 29].

Despite the importance of motor unit behavior for explosive performance [32], no studies have determined whether sex differences in MUDR exist. Given the greater proportion of type I relative to type II fibers area in muscles of females than males (~20% in tibialis anterior muscle; [10, 11]), with slower contractile kinetics [33], the recruitment rate of motor units and their discharge rate in explosive efforts may be higher to compensate for the slower muscle contractile properties. A similar mechanism has been recently pointed out for chronically strength‐trained individuals [34], whereas resistance‐trained compared to untrained participants showed higher MUDR at the onset of rapid contractions, possibly to partly compensate for the training‐induced slowing of muscle contractile properties.

The present research aimed to study sex differences in rapid torque production of voluntary and electrically evoked explosive contractions of the ankle dorsiflexors. During voluntary explosive efforts, the firing rate of individual motor units was assessed via decomposition of high‐density EMG signals recorded from the tibialis anterior muscle. Based on the evidence presented above, we hypothesized comparable and equivalent values (verified with equivalence statistics) of relative RTD measures between sexes (i.e., when accounted for MVT), with higher values of MUDR in females than males.

## 2 Methods

### 2.1 Ethics Statement

All testing procedures were approved by the Ethics Commission of the University of Konstanz (Institutional Review Board Statement 10/2023) and conformed to the standards set by the *Declaration of Helsinki*, except for registration in a database.

### 2.2 Participants

Thirty‐four recreationally active young adults (22 female; mean ± standard deviation; age: 27 ± 6 years; height: 170 ± 4 cm; body mass: 61 ± 6 kg and 12 male; 26 ± 4 years; 181 ± 7 cm; 85 ± 15 kg) participated in the study after providing informed written consent. Before measurements, females self‐reported their average menstrual cycle duration, the day of the last ovulation, and whether they took oral contraceptives.

**FIGURE 1:**
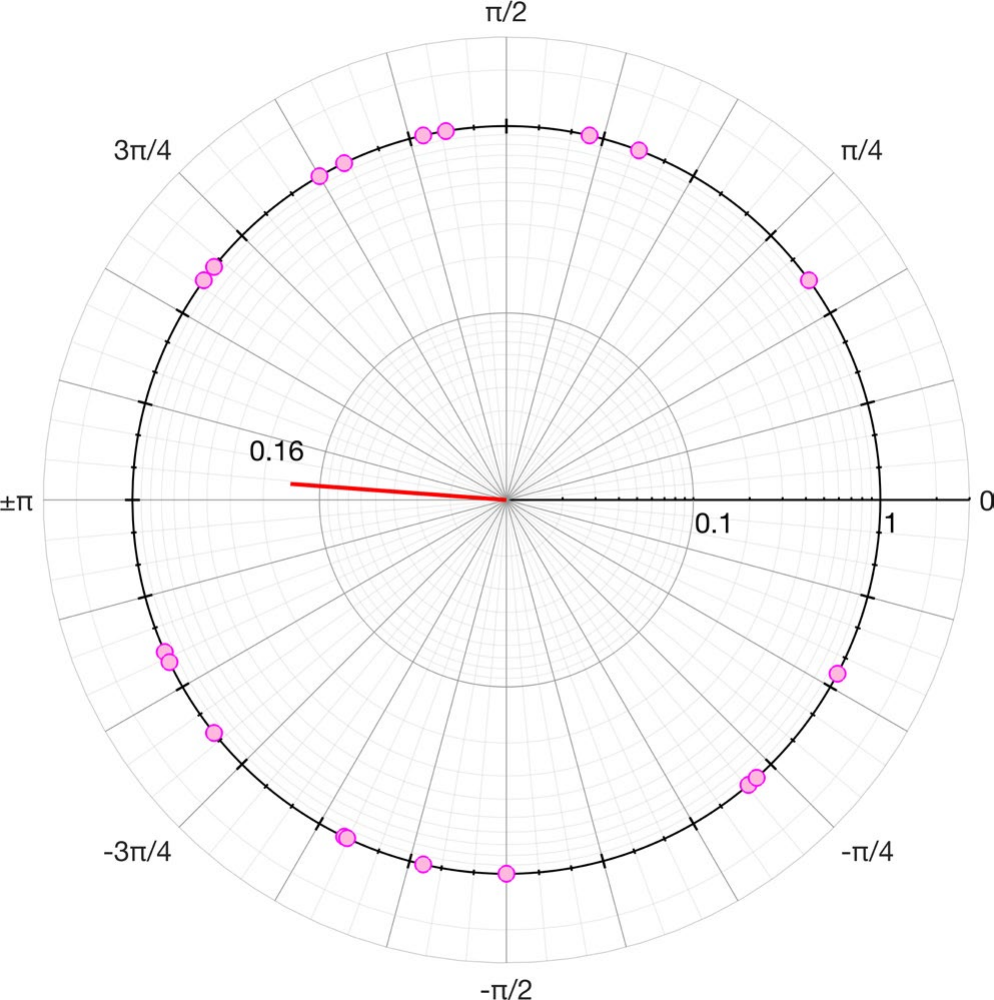
Polar‐log diagram representing the last ovulation day from experimental testing relative to the average self‐reported cycle duration. Pink circles represent individual data points. The red line represents the mean resultant vector with magnitude 0.16. The circular distribution was checked for uniformity using the Rayleigh test and modeled with a von Mises probability function. The high *p* value from the Rayleigh test (0.63), the low resulting κ value of the modeled von Mises distribution (0.32), and the direction and low magnitude of the mean resultant vector (3.07 rad and 0.16, respectively) indicated that the experimental sessions were not skewed toward any specific self‐reported phase of the menstrual cycle.

**FIGURE 2:**
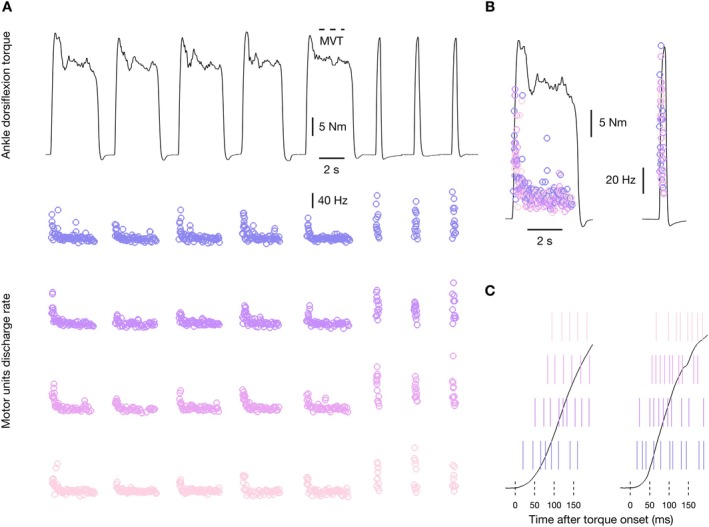
Exemplar of data analysis of the high‐density EMG signal from one female participant. (A) The three explosive‐impulse and three explosive‐hold contractions selected (based on RTD in the window 0–150 ms from torque onset), in addition to further 2 explosive‐hold efforts were concatenated and decomposed (see the Section 2.7.2 for details). Identified motor unit firings were edited, and only motor units with a reliable discharge pattern and a pulse‐to‐noise ratio ≥ 30 dB were retained [43, 44]. In this female participant, 4 motor units (out of 8 identified) were retained, and circles represent their instantaneous discharge rate. (B) Torque in the first explosive‐hold and explosive‐impulse contractions of those concatenated, with the instantaneous discharge rate of the retained motor units pooled together. (C) Raster plot of individual motor unit discharges in the range −25 to 200 ms from torque onset. Note that discharges of the retained motor units could be identified only after the onset of torque.

The number of females recruited was purposely defined a priori as twice that of males for two reasons: (1) to increase the likelihood of testing female participants evenly across the self‐reported phases of the menstrual cycle (see Figure 1); (2) to account for the nearly half yield of motor units number from the tibialis anterior muscle typically found in females relative to males [17], which worsens the probability that values of MUDR calculated from the identified motor units represent the mean firing rate of the entire active motor units pool [35]. This solution, however, does not solve the limitation that detected motor units from high‐density EMG in females may tend to be of higher activation threshold than males due to filtering effects [17].

### 2.3 Study Design

Participants were tested on two separate occasions. Session 1 regarded familiarization with isometric maximal as well as explosive contractions of the ankle dorsiflexors. This muscle group was selected due to the ease of motor unit identification in both sexes, and as explosive contractions can be evoked by supramaximal electrical stimulation of the common fibular nerve with minimal discomfort for participants. For the explosive contractions, participants were instructed to avoid any countermovement. Session 2 (performed at least 48 h after session 1) included isometric maximal voluntary, electrically evoked, and explosive contractions of the ankle dorsiflexors.

### 2.4 Experimental SetUp

Participants sat on an isokinetic dynamometer (Isomed 2000; Ferstl GmbH, Hemau, Germany). The right foot was placed on a custom‐made footplate at 30° plantarflexion and secured with Velcro straps across the instep and toes. A 90° angle at the knee joint was ensured, with the thigh strapped to a C‐clamp to avoid thigh movements during dorsiflexor contractions. Ankle dorsiflexion torque was measured by a torque transducer (DV‐14, Lorenz Messtechnik GmbH, Alfdorf, Germany) connected to the custom‐made footplate. The output signal was amplified (x1000, LCV/U10, Lorenz Messtechnik, GmbH), low‐pass filtered (cutoff frequency: 1000 Hz; Neurolog System NL530 module; Digitimer, Welwyn Garden City, United Kingdom), sampled at 10 000 Hz using a 16‐bit A/D converter (CED 1401‐3; Cambridge Electronic Design, Cambrige, United Kingdom), and recorded using Spike 2 software (v. 8.2; Cambridge Electronic Design). With this setup, the three standard deviations of the baseline torque were 0.08 Nm, that is, 0.2% MVT considering a realistic ankle dorsiflexion MVT of 40 Nm (see fig. 6 in [1] for comparison). Participants were always provided with visual torque feedback. The analog torque signal was also fed to a 16‐bit multichannel amplifier (Sessantaquattro+, OT Bioelettronica, Turin, Italy) and sampled at 2000 Hz for synchronization with high‐density EMG recordings (see below) using OT Biolab Software (OT Bioelettronica). In session 2, high‐density EMG signals were recorded from the tibialis anterior muscle. The skin was shaved, lightly abraded, and cleaned with a skin preparation solution (Everi, Spes Medica, Battipaglia, Italy). An adhesive grid of 64 electrodes (13 rows × 5 columns; electrode diameter: 1 mm; interelectrode distance: 8 mm; HD08MM1305, OT Bioelettronica) was then placed over the tibialis anterior muscle according to the guidelines from SENIAM [36], with the orientation of the major grid axis aligned with the major axis of the muscle. The adhesive grid was held on the skin using a semi‐disposable bi‐adhesive foam layer (Spes Medica). The skin‐electrode contact was made by filling the cavities of such foam layer with conductive paste (AC Cream, Spes Medica). The grid electrodes were further fixed on the skin by adhesive tape. A strap electrode (reference electrode) was dampened with water and placed around the ipsilateral ankle. Signals from high‐density EMG were acquired in monopolar mode, bandpass filtered (10–500 Hz), sampled at 2000 Hz, and recorded via a 16‐bit multichannel amplifier (Sessantaquattro+, OT Bioelettronica) using OT Biolab Software (OT Bioelettronica). In addition to high‐density EMG, the surface EMG activities of tibialis anterior, soleus, gastrocnemius medialis, and lateralis were recorded using a bipolar configuration (1‐cm inter‐electrode distance; Trigno System; Delsys, Boston, MA). The guidelines from SENIAM were followed for electrode location except for tibialis anterior, for which the electrode was placed distally to the high‐density EMG matrix. The surface bipolar EMG signals were amplified and band‐pass filtered (x300, 20–450 Hz), and sampled at 2000 Hz (CED 1401‐3; Spike2 software). The surface bipolar EMG system presented an intrinsic constant delay relative to the torque signal. Such delay was measured (in pilot testing with electrical stimulation) as the time between an electrical stimulus trigger and the onset of the stimulus artifact in the EMG signal and quantified as 59 ms. This value was invariant across muscle groups (ankle dorsiflexors and plantar flexors, knee extensors, elbow flexors and extensors) and participants. Thus, for all off‐line analyses, the surface bipolar EMG signals were shifted leftward to correct for time delay.

### 2.5 Common Fibular Nerve Stimulation

Square‐wave electrical stimuli (pulse width: 500 μs; 100–400 V) were delivered to the common fibular nerve using a constant current stimulator (DS7A; Digitimer Ltd). A bar electrode (279‐930‐24TP; Chalgren Enterprises, Gilroy, Canada) was held in place by the operator distal and posterior to the fibular head. Stimulus intensity was set at 120% of the current required to obtain the maximal resting twitch peak torque of the dorsiflexors, ensuring supramaximal stimulation for all electrically evoked contractions (15–70 mA).

### 2.6 Experimental Procedures

Data collection (session 2) began with the determination of the stimulation intensity for the maximal resting twitch peak torque of the dorsiflexors and for the resting maximal compound muscle action potential (*M*_max_). Stimulus intensity was raised incrementally until the peak torque of the dorsiflexors resting twitch reached a plateau. Intensity was then increased by 20%, and two resting stimuli were delivered.

Six isometric electrically evoked contractions were then performed, consisting of three trains of 7 pulses at 10 Hz and of 50 pulses at 200 Hz, every 30 s. Seven pulses were specifically chosen because the peak torque of 10‐Hz trains is achieved by the 7th pulse [37, 38]. The 200‐Hz trains of stimuli were performed to emulate rapid contractions of the ankle dorsiflexors; such frequency was chosen after pilot testing as yielding the greatest peak rate of torque development (pRTD), and the number of pulses (50) for the contractions to last approximately as long as an explosive impulsive effort.

Participants then performed two brief (~3 s; 90 s rest in‐between) isometric maximal voluntary contractions to establish ankle dorsiflexion MVT. Additional contractions were performed if MVT changed by ≥ 5% with successive contractions. After the determination of MVT, participants were asked to perform 10 explosive impulsive contractions, with 30 s of rest in‐between.

For these rapid contractions, hereafter referred to as explosive‐impulse, participants were instructed to contract “as fast as possible,” producing the greatest impulse in the shortest time.

From the maximal isometric contractions, MVT was then used to set a torque target on the computer monitor at 75% MVT. Participants then performed two sets with 2 min of rest in‐between five rapid contractions, hereafter referred to as explosive‐hold, interspersed by 1‐min rest, with the instruction to reach the target torque as fast as possible and hold it for ~3 s [39, 40].

Finally, participants performed 2 maximal isometric contractions (90s rest in‐between). Pre‐to‐post protocol changes in MVT were −0.2 ± 3.6% across all participants, indicating no changes in muscle performance from the experimental protocol.

### 2.7 Data Analysis

#### 2.7.1 Torque and EMG

All signals were analyzed off‐line using Spike (v. 8.2) or custom MATLAB scripts (v2023b; MathWorks, Natick, MA, USA). Analyses regarding the root‐mean‐square (RMS) of the EMG signal and the peak‐to‐peak amplitude of the *M*_max_ were performed on data acquired from both the surface bipolar EMG and monopolar high‐density EMG systems. For the high‐density EMG, channels with poor signal‐to‐noise ratio (baseline noise peak‐to‐peak > 50 μV) were removed based on visual inspection using a semi‐automated MATLAB script. Given the congruency of the results between the two EMG systems, only high‐density EMG data are here presented, with surface bipolar EMG results reported in the Supporting Information S2 and in Tables S7 and S8. The peak‐to‐peak amplitude of the *M*_max_ was calculated from each high‐density EMG channel and then averaged within the same subject to obtain a representative M‐wave amplitude of the muscle. Peak torque was obtained from each train of stimuli and averaged separately for 10 Hz and 200 Hz. Electrically evoked rapid contractions were completed only from 13 females and 10 males.

Channels from the monopolar high‐density EMG system (after removal of dc offset, and rectification) were averaged within the same subject to obtain a representative muscle activity signal. The onset of torque (for evoked and voluntary explosive contractions) and EMG (for voluntary contractions only) were then identified using a hybrid automatic‐manual procedure with MATLAB. The first automatic procedure allowed for quickly selecting the region of interest close to torque onset for each rapid contraction. First, a Butterworth fourth‐order lowpass filter (cutoff frequency: 500 Hz) was applied. Torque was then downsampled from 10 000 to 2000 Hz. For each rapid contraction, RTD of a 10‐ms moving window was calculated, and the peak value (pRTD) was identified. The torque between 600 and 400 ms before pRTD was averaged to represent baseline. The temporary torque onset was then determined moving backward, as the last through after the first instant when RTD was greater than 5 Nm s^−1^ and torque was above 2 standard deviations from baseline. The temporary torque onset was visualized for each contraction, and “true” torque onset was manually identified following gold‐standard procedures [41]. That is, the torque signal was visualized 40 ms before and 10 ms after the temporary onset, in a 0.6 Nm range, and the torque onset could be modified when needed as the last through before a clear upward deflection of the signal from baseline. For each rapid contraction (evoked and voluntary), the RTD function was calculated, that is, the derivative of torque with increasing (0.5 ms steps) time interval from torque onset, from 0 to 150 ms. The high‐density EMG average signal from the tibialis anterior muscle was then represented for each explosive contraction 500 ms around torque onset. The onset of EMG was manually identified similar to torque, as the last through before a clear upward deflection from baseline [41]. For all rapid contractions (evoked and voluntary), RTD in the windows 0–50, 0–100, and 0–150 ms from torque onset (from the RTD function) and impulse (IMP) were determined. The maximal value of the RTD function (pRTDf) was determined, and the time to achieve both pRTD and pRTDf from torque onset were calculated. For voluntary contractions only, RMS EMG was determined in the windows 0–50, 0–100, and 0–150 ms after EMG onset. The three explosive‐impulse and three explosive‐hold contractions showing the greatest RTD within 0–150 ms from torque onset were considered for further analyses. This time window was selected as both pRTD and pRTDf are typically reached within 150 ms in all participants.

All EMG‐related measures were normalized to the *M*_max_ peak‐to‐peak amplitude. Torque‐related measures for 200‐Hz trains, explosive‐impulse, and explosive‐hold contractions are reported as raw values and normalized to MVT (Tables 1 and 2, Figures 4 and 5).

**TABLE 1:** Absolute torque variables in males and females in explosive‐impulse and explosive‐hold contractions, and 200‐Hz trains.

Variables	Explosive‐impulse		Explosive‐hold		200‐Hz train	
	Males	Females	Males	Females	Males	Females
pRTD (Nm s^−1^)	331 ± 86	215 ± 33	292 ± 49	206 ± 31	280 ± 51^1^	167 ± 53^1^
pRTDf (Nm s^−1^)	201 ± 34	141 ± 25	187 ± 39	137 ± 22	195 ± 28	132 ± 37
RTD (Nm s^−1^)						
0–50 ms	131 ± 43	91 ± 30	109 ± 45	83 ± 26	193 ± 30^2^	121 ± 33^2^
0–100 ms	195 ± 35	137 ± 25	179 ± 44	131 ± 24	167 ± 29^1^	99 ± 29^1^
0–150 ms	187 ± 28	132 ± 21	181 ± 35	132 ± 21	137 ± 26^1^	79 ± 24^1^
IMP (Nm s)						
0–50 ms	0.11 ± 0.04	0.08 ± 0.03	0.09 ± 0.04	0.07 ± 0.02	0.21 ± 0.04^2^	0.14 ± 0.04^2^
0–100 ms	0.77 ± 0.17	0.54 ± 0.13	0.68 ± 0.21	0.50 ± 0.12	0.89 ± 0.14^2^	0.55 ± 0.16^2^
0–150 ms	1.98 ± 0.35	1.39 ± 0.26	1.85 ± 0.45	1.34 ± 0.25	1.84 ± 0.31^1^	1.10 ± 0.31^1^

*Note:* Values are the mean ± standard deviation. When accounting for MVT, no differences between sexes were evident (Table S1), but torque‐related variables were lower or shorter (^1^), or higher (^2^), in electrically evoked contractions.

Abbreviations: IMP, torque impulse; pRTD, peak rate of torque development; pRTDf, peak rate of torque from the RTD function.

**TABLE 2:** Relative torque variables and muscle electrical activity in males and females in explosive‐impulse and explosive‐hold contractions, and 200‐Hz trains.

Variables	Explosive‐impulse		Explosive‐hold		200‐Hz train	
	Males	Females	Males	Females	Males	Females
Normalized pRTD (MVT s^−1^)	8.0 ± 2.0	7.7 ± 0.8	7.1 ± 1.1	7.3 ± 0.7	6.4 ± 0.7^1^	6.1 ± 2.1^1^
Normalized pRTDf (MVT s^−1^)	4.8 ± 0.6	5.0 ± 0.6	4.5 ± 0.7	4.9 ± 0.7	4.5 ± 0.6	4.6 ± 1.5
Time to pRTD (ms)	61 ± 12**	74 ± 14	71 ± 16**	79 ± 15	24 ± 3**^,1^	28 ± 7^1^
Time to pRTDf (ms)	115 ± 24	113 ± 18	128 ± 22	125 ± 20	44 ± 11^1^	42 ± 18^1^
Normalized RTD (MVT s^−1^)						
0–50 ms	3.1 ± 0.8	3.2 ± 0.9	2.6 ± 0.8	3.0 ± 0.8	4.4 ± 0.6^2^	4.4 ± 1.4^2^
0–100 ms	4.7 ± 0.7	4.9 ± 0.6	4.3 ± 0.7	4.7 ± 0.7	3.8 ± 0.4^1^	3.6 ± 1.2^1^
0–150 ms	4.5 ± 0.4	4.7 ± 0.4	4.3 ± 0.5	4.7 ± 0.4	3.1 ± 0.3^1^	2.9 ± 0.9^1^
Normalized IMP (MVT s)						
0–50 ms	0.003 ± 0.001	0.003 ± 0.001	0.002 ± 0.001	0.003 ± 0.001	0.005 ± 0.001^2^	0.005 ± 0.002^2^
0–100 ms	0.019 ± 0.004	0.019 ± 0.004	0.016 ± 0.004	0.018 ± 0.004	0.021 ± 0.002^2^	0.020 ± 0.004^2^
0–150 ms	0.048 ± 0.007	0.049 ± 0.006	0.043 ± 0.007	0.048 ± 0.007	0.042 ± 0.004^1^	0.040 ± 0.005^1^
RMS EMG (A.U.)						
0–50 ms	0.071 ± 0.029	0.077 ± 0.040	0.062 ± 0.024	0.062 ± 0.035		
0–100 ms	0.090 ± 0.026	0.100 ± 0.039	0.076 ± 0.021	0.085 ± 0.029		
0–150 ms	0.096 ± 0.019	0.109 ± 0.041	0.084 ± 0.020	0.096 ± 0.028		

*Note:* Values are the mean ± standard deviation. No sex‐related differences were found for all variables, except for time to pRTD, that across all contraction types was shorter in males than females (**p < 0.01). After accounting for MVT, torque‐related variables were lower or shorter (^1^), or higher (^2^), in electrically evoked contractions.

Abbreviations: IMP, torque impulse; pRTD, peak rate of torque development; pRTDf, peak rate of torque from the RTD function; RMS EMG, root‐mean‐square from the high‐density electromyography signal.

To have greater insights into the voluntary capacity to produce torque and impulse rapidly, voluntary evoked ratios were calculated by dividing pRTD, time to pRTD, pRTDf, time to pRTDf, RTD, and IMP in the three time windows between voluntary explosive contractions and 200‐Hz trains.

#### 2.7.2 High‐Density EMG Decomposition

From the high‐density EMG, values of MUDR were identified following standard procedures [34, 39, 40]. Briefly, monopolar high‐density EMG signals were band‐pass filtered (Butterworth 5th order, zero lag, 20–500 Hz), channels with poor signal‐to‐noise ratio and movement artifacts were removed, and signals were decomposed into motor units spike trains using the Convolution Kernel Compensation Algorithm [42]. The three explosive‐impulse and three explosive‐hold contractions selected (based on RTD in the window 0–150 ms from torque onset), in addition to a further two explosive‐hold efforts (to increase the contraction duration spent at the 75% MVT plateau) were concatenated and decomposed. Identified motor unit firings were edited, and only motor units with a reliable discharge pattern and a pulse‐to‐noise ratio ≥ 30 dB were retained following standard procedures [43, 44]. An exemplar of data analysis of the high‐density electromyography signal from one female participant is reported in Figure 2. Given that MUDR across the first few pulses is an important determinant of explosive strength, following recently used procedures [34], the discharge rate at the beginning of explosive‐impulse and explosive‐hold contractions was determined as the average of the reciprocal of the first three interspike intervals. Notably, only discharges after the onset of torque for the selected motor units could be identified (see Figure 2C). In addition, MUDR was also determined in the plateau phase of explosive‐hold contractions, considering the 10 interspike intervals at 0.5 s from contraction onset. For each motor unit, the average discharge rate was calculated with a 35‐ms window moving in 1‐ms steps for 500 ms. The resulting functions were averaged within sexes, yielding mean MUDR functions for males and females.

### 2.8 Statistics

Statistical analysis was conducted using MATLAB. For all comparisons, linear mixed‐effects models were used (*fitlme* function), fitted with maximum likelihood estimation. After checking normality of residuals (normality test package; [45]), baseline values of MVT and *M*_max_ peak‐to‐peak amplitude were compared between sexes (Dependent variable ~1 + Sex). Torque‐related measures and RMS EMG between sexes within voluntary and electrically evoked contractions were compared (Dependent variable ~1 + Sex · MVT), with *M*_max_ peak‐to‐peak amplitude as a fixed factor instead of MVT for EMG‐related variables. To verify whether explosive strength was equivalent between sexes, all RTD‐related measures were expressed relative to MVT and compared within contraction types using two one‐sided hypothesis testing [46]. Equivalence tests are dependent on the choice of the smallest effect size that is not considered practically meaningful: the wider its value, the wider the equivalence bounds, and the greater the likelihood of finding equivalent results [47]. Given that several torque‐related variables were compared, we opted to choose equivalence bounds based on a moderate relative effect size of 0.6 (Cohen's *d*). To put this into context, equivalence bounds for the difference in relative pRTD in explosive‐impulse contractions were ± 0.842 MVT s^−1^; the upper equivalence bound represented half of the improvement in relative pRTD after a 3‐month explosive training of the ankle dorsiflexors (~1.7 MVT s^−1^; [48]). Given the absence of sex‐related differences when accounting for the variance explained by MVT and *M*_max_ peak‐to‐peak amplitude, torque‐related variables, and RMS EMG were compared between contractions (explosive‐impulse, explosive‐hold contractions, and 200‐Hz trains) pooling data between sexes (Dependent variable ~1 + MVT + Contraction type + (1 | Participant); with *M*_max_ peak‐to‐peak amplitude instead of MVT as fixed factor for comparison of RMS EMG between explosive‐impulse and explosive‐hold contractions). Time to pRTD and pRTDf between all contraction types, and MUDR at the beginning of explosive‐impulse and explosive‐hold contractions were then examined (Dependent variable ~1 + Sex · Contraction type + (1 | Participant)). For the comparison of MUDR in the plateau phase of explosive‐hold contractions between sexes, only the fixed factor of sex was kept within the model (Dependent variable ~1 + Sex). Assumptions for the linear mixed‐effects models (linearity, independence of residual errors, normality of residuals, and residuals variance) were checked for each dependent variable. Since no significant Sex · MVT, Sex · *M*_max_ peak‐to‐peak amplitude, or Sex · Contraction type interactions were found, statistical regressions were rerun, including fixed factors but removing the interactions from the model equations. The significance level was set at *p* < 0.05. Outcomes from the linear mixed‐effects models are reported in the section S1 of the Supporting Information and in Tables S1–S3. Outcomes from the equivalence tests are reported in Tables S4–S6.

## 3 Results

### 3.1 Participants Characteristics

Of the 22 females, 19 reported to be eumenorrheic (regular cycle between 21 and 35 days for the previous 12 months; [49]), and 6 had taken oral contraceptives within the previous 12 months. The distribution of the self‐reported last ovulation day from experimental testing relative to the average self‐reported cycle duration (in polar‐log coordinates in Figure 1) was checked for circular uniformity using the Rayleigh test ([50]; using MATLAB), and the circular distribution was modeled with a von Mises probability function [51]. The high *p* value from the Rayleigh test (0.63), the low resulting κ value of the modeled von Mises distribution (0.32), and the direction and low magnitude of the mean resultant vector (3.07 rad and 0.16, respectively; red line in Figure 1) indicated that the experimental sessions were not skewed toward any specific self‐reported phase within the menstrual cycle.

### 3.2 Maximal Torque and *M*_max_ Peak to Peak Amplitude

MVT (males: 41.9 ± 7.0 Nm; females: 28.2 ± 4.1 Nm) and *M*_max_ peak‐to‐peak amplitudes (males: 8.7 ± 2.0 mV; females: 6.6 ± 1.8 mV) were higher in males than in females (Supporting Information S1). When accounting for MVT, no differences between groups were evident for the peak torques of 10 Hz (males: 11.9 ± 4.3 Nm; females: 8.1 ± 2.5 Nm) and 200‐Hz trains (males: 26.0 ± 4.9 Nm; females: 14.6 ± 4.4 Nm; Table S1).

### 3.3 Explosive Contractions

**FIGURE 3:**
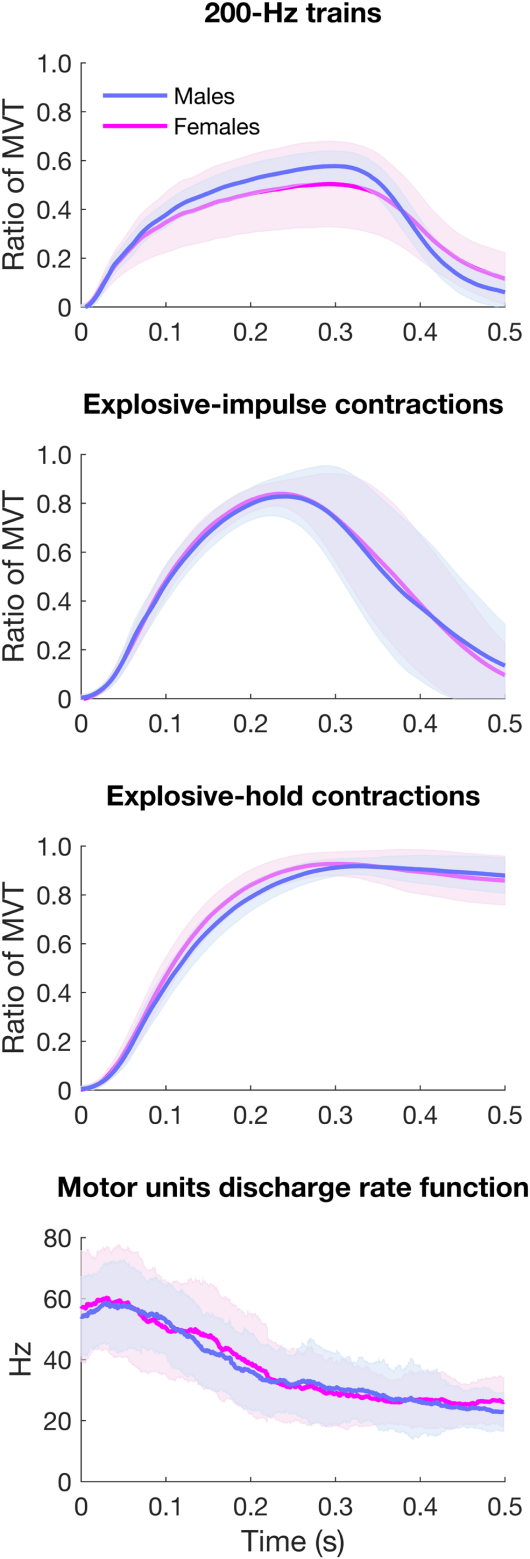
Mean torque (relative to MVT) of the 200‐Hz trains, explosive‐impulse and explosive‐hold contractions, as well as mean motor units discharge rate function in explosive‐hold contractions, in males (blue) and females (magenta). The discharge rate function was calculated for each motor unit with a 35‐ms window moving in 1‐ms steps.

**FIGURE 4:**
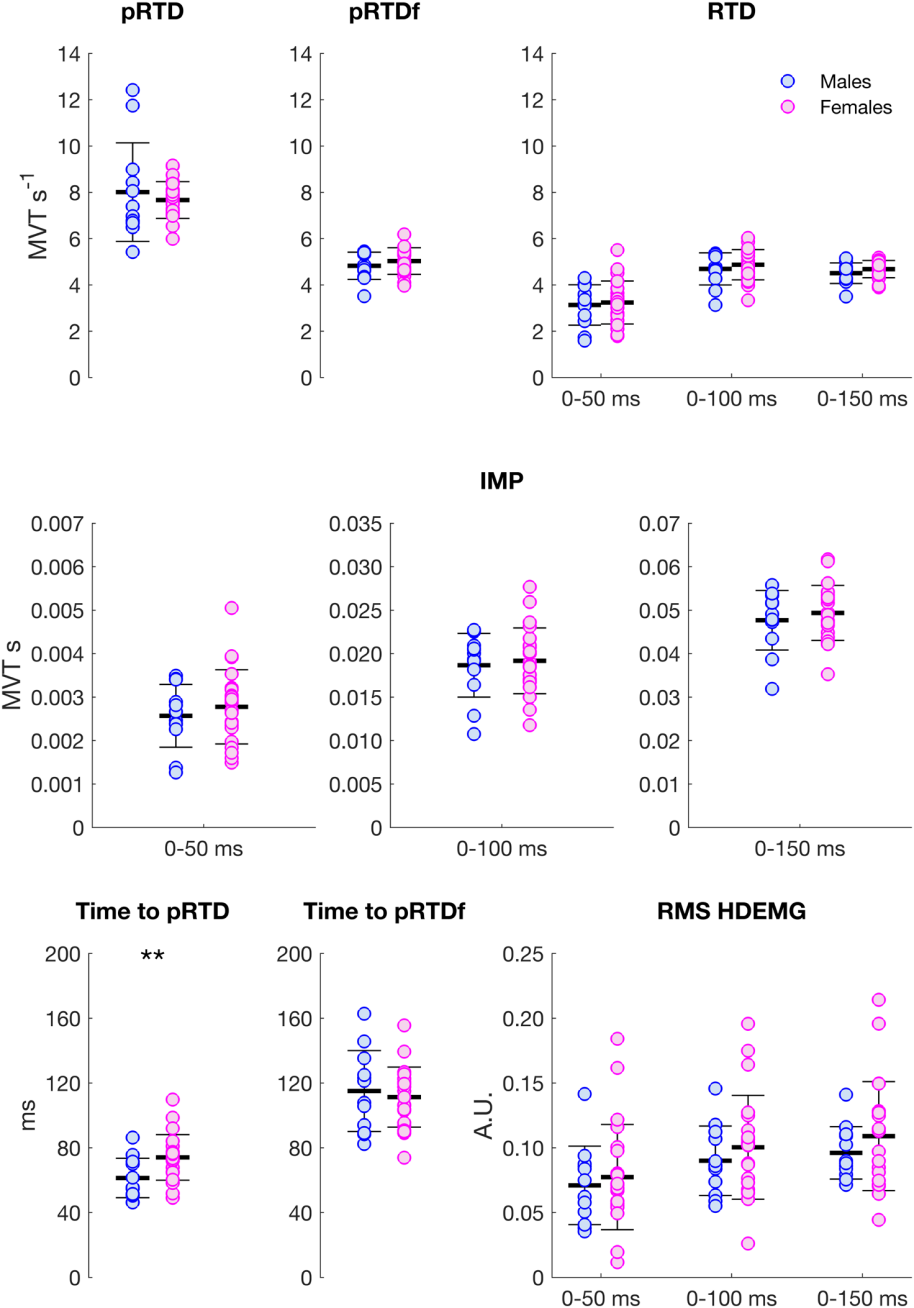
Relative torque and RMS EMG variables (normalized to MVT and *M*_max_ peak‐to‐peak amplitude, respectively) in males (blue) and females (magenta) in explosive‐impulse contractions. Bars represent means and standard deviations, whereas circles represent individual values. pRTD, peak rate of torque development; pRTDf, peak rate of torque from the RTD function; IMP, torque impulse; RMS EMG, root‐mean‐square from the high‐density electromyography signal. No differences were found between sexes, except for time to pRTD, which was shorter in males than females (**; Table S3).

Mean torque traces divided by sex for 200‐Hz trains, explosive‐impulse, and explosive‐hold contractions are reported in Figure 3. Absolute RTD and IMP values for explosive‐impulse and explosive‐hold contractions, and 200‐Hz trains are reported in Table 1. Relative torque measures (normalized to MVT) and RMS EMG (normalized to *M*_max_ peak‐to‐peak amplitude) are reported in Table 2, and in Figure 4 for explosive‐impulse contractions only. For all measures, MVT and *M*_max_ peak‐to‐peak amplitude were significant predictors. When the effect of such predictors was accounted for, no sex‐related differences were found (Table S1). The only exception was time to pRTD, which was shorter across all contraction types in males compared to females between 4 and 18 ms (Table S3).

None of the torque‐related measures normalized to MVT were equivalent between sexes (Tables S4–S6). Therefore, relative RTD measures were neither statistically different nor equivalent between sexes.

### 3.4 Differences Between Contraction Types

**FIGURE 5:**
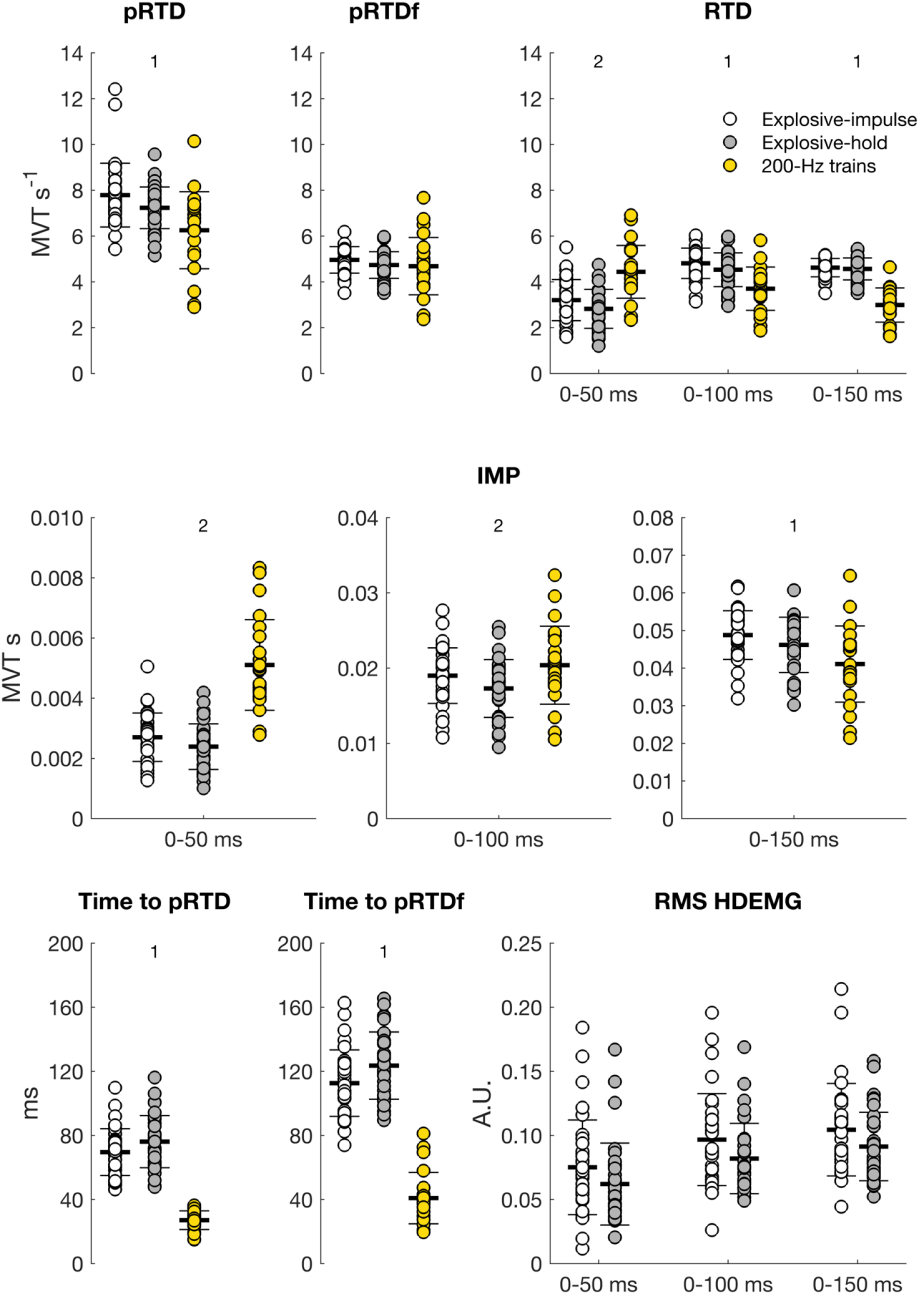
Relative torque and RMS EMG variables (normalized to MVT and M_max_ peak‐to‐peak amplitude, respectively), pooled between sexes, in explosive‐impulse (white) and explosive‐hold (gray) contractions, and in 200‐Hz trains (yellow). Bars represent means and standard deviations, whereas circles represent individual values. IMP, torque impulse; pRTD, peak rate of torque development; pRTDf, peak rate of torque from the RTD function; RMS EMG, root‐mean‐square from the high‐density electromyography signal. No significant differences were found for any of the variables between explosive‐impulse and explosive‐hold contractions. Electrically evoked compared to voluntary contractions yielded higher values of RTD in the window 0–50 ms, and IMP in the windows 0–50 and 0–100 ms from contraction onset (^2^), while lower values were reported for all other torque‐related variables, along with shorter time to pRTD and to pRTDf (^1^; Table S2).

Explosive relative torque measures and RMS EMG for all contraction types, pooled between sexes, are reported in Figure 5. The voluntary:evoked ratios for explosive‐impulse and explosive‐hold contractions are reported in Table 3. No significant differences were found for any of the variables between explosive‐impulse and explosive‐hold contractions. Electrically evoked compared to voluntary contractions yielded higher values of RTD in the window 0–50 ms, and higher IMP in the windows 0–50 and 0–100 ms from contraction onset. However, electrically evoked contractions reported lower values for all other torque‐related variables and windows, and shorter time to pRTD, but no differences with voluntary contractions in time to pRTDf (Table S2).

**TABLE 3:** Voluntary:evoked ratios for torque‐related variables in explosive‐impulse and explosive‐hold contractions.

Variables	Explosive‐impulse		Explosive‐hold	
	Males	Females	Males	Females
Normalized pRTD	1.17 ± 0.21	1.40 ± 0.56	1.07 ± 0.18	1.33 ± 0.53
Normalized pRTDf	1.05 ± 0.12	1.11 ± 0.37	1.01 ± 0.14	1.10 ± 0.39
Time to pRTD	2.11 ± 0.45	3.31 ± 1.00	2.35 ± 0.44	3.46 ± 1.25
Time to pRTDf	2.82 ± 0.80	3.64 ± 1.26	3.03 ± 0.85	3.87 ± 1.47
Normalized RTD				
0–50 ms	0.72 ± 0.18	0.74 ± 0.29	0.62 ± 0.18	0.70 ± 0.29
0–100 ms	1.20 ± 0.19	1.43 ± 0.50	1.14 ± 0.19	1.39 ± 0.48
0–150 ms	1.43 ± 0.18	1.76 ± 0.59	1.40 ± 0.18	1.77 ± 0.57
Normalized IMP				
0–50 ms	0.52 ± 0.13	0.54 ± 0.23	0.46 ± 0.14	0.52 ± 0.23
0–100 ms	0.89 ± 0.16	0.98 ± 0.34	0.82 ± 0.17	0.93 ± 0.35
0–150 ms	1.11 ± 0.15	1.29 ± 0.44	1.06 ± 0.17	1.26 ± 0.42

*Note:* Values are the mean ± standard deviation.

Abbreviations: IMP, torque impulse; pRTD, peak rate of torque development; pRTDf, peak rate of torque from the RTD function.

### 3.5 Motor Units Discharge Rate

**FIGURE 6:**
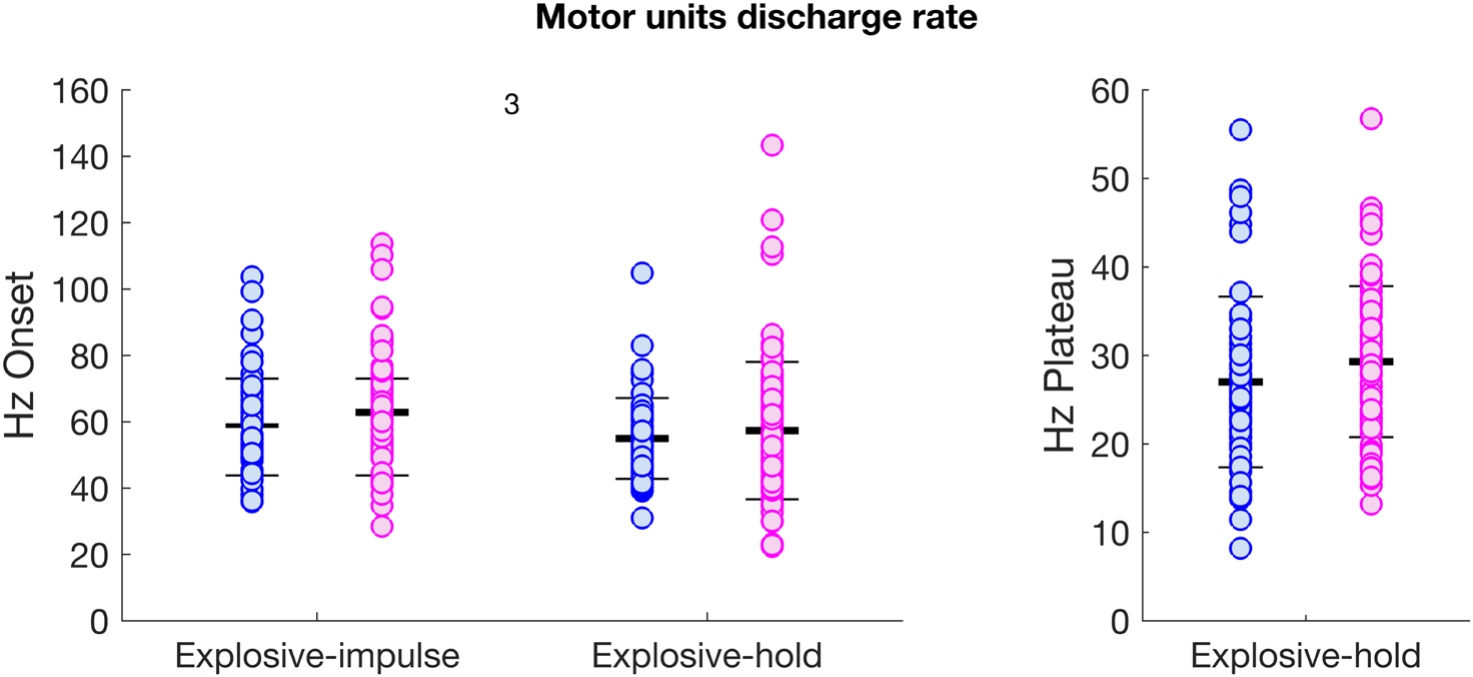
Motor units discharge rate at the onset of explosive‐impulse and explosive‐hold contractions (left), and in the plateau phase of explosive‐hold contractions (right), in males (blue) and females (magenta). Bars represent means and standard deviations, whereas circles represent individual values. No significant differences were found between sexes. Values of motor units discharge rate were significantly higher (~6 Hz) at the beginning of explosive‐impulse compared to explosive‐hold contractions irrespective of sex (^3^; Table S3).

The number of identified motor units in females was 87 (~4 ± 2 per participant), versus 71 identified in males (~6 ± 2 per participant, respectively). The mean motor unit discharge rate function divided by sex in explosive‐hold contractions is reported in Figure 3. Discrete values of MUDR at the onset of explosive‐impulse and explosive‐hold contractions, and in the plateau phase of explosive‐hold contractions are reported in Figure 6. No differences were found between sexes. Values of MUDR were significantly higher (difference of ~6 Hz) at the beginning of explosive‐impulse compared to explosive‐hold contractions irrespective of sex (Figure 6, Table S3).

## 4 Discussion

The main findings of the present study were as follows: (1) when accounting for MVT and *M*_max_ peak‐to‐peak amplitude, rapid torque production and muscle activity were not significantly different between sexes; (2) despite the lack of significant differences, normalized RTD and IMP metrics were not equivalent between sexes; (3) the time to pRTD from contraction onset, but not the time to pRTDf, was shorter in males than females; (4) compared to voluntary contractions, electrically evoked contractions showed a shorter time to pRTD, with higher RTD and impulse in the early phase following torque onset. In contrast, during the later phase, they exhibited lower RTD and impulse values; (5) values of MUDR at the beginning of the explosive contractions and in the plateau phase were not different between sexes. Thus, despite subtle differences in the kinetics of explosive contractions, rapid torque production and the associated motor unit behavior were not different between males and females.

### 4.1 Explosive Strength Between Sexes

Males demonstrated greater maximal torque than females during maximal voluntary contractions, and in response to 10‐ and 200‐Hz supramaximal trains of stimuli. This difference and its magnitude (maximal torque of females as percentage of that of males: 60%–65%) are consistent with earlier reports [9], and have been attributed mainly to a larger muscle mass, greater fiber cross‐sectional area, and a higher type II/type I muscle fiber area ratio in men compared to women [10, 11, 27, 52]. When MVT was accounted for, no differences between sexes were evident in the maximal ankle dorsiflexion torque of 10 and 200‐Hz trains, consistent with previous work [53].

The *M*_max_ peak‐to‐peak amplitude was higher in males compared to females. This can be attributed to the larger cross‐sectional area of muscle fibers in males, which results in motor unit potentials of greater amplitude [17, 20, 52]. The synchronous summation of these higher amplitude motor unit potentials likely contributes to the greater peak‐to‐peak amplitude of the *M*_max_ observed in males. Indeed, previous research indicates that the *M*_max_ amplitude is sensitive to the amount of muscle cross‐sectional area underneath the recording electrode. For instance, the amplitude of the *M*_max_ was higher in moderately trained and long‐term strength trained individuals compared to untrained participants [54, 55], and is typically reduced in older adults, due to age‐related declines in the excitable muscle mass [56]. There are, however, exceptions to these observations. Despite having lower muscle mass, females exhibited comparable *M*_max_ peak‐to‐peak amplitude to males in the vastus lateralis and rectus femoris [27]. Similarly, no difference in the *M*_max_ amplitude was observed in the biceps brachii of Sherpas compared to Lowlanders [57], or in the case of muscle unloading‐related atrophy [58].

All absolute torque‐related measures of rapid torque production were greater in males than females in the present study. When MVT was accounted for in the linear regression models, sex differences were abolished. This corroborates findings from previous studies with measures of explosive strength and muscle activity between sexes from elbow flexors [25, 26], knee flexors and extensors [27, 28, 29], and ankle dorsiflexors [30, 31]. Values of pRTD, pRTDf, as well as RTD in the windows 0–50, 0–100, and 0–150 ms from onset are consistent with those reported from earlier research from ankle dorsiflexors [39, 59].

Given that the statistical analysis with traditional null‐hypothesis testing is informative only of rejection of the null hypothesis (i.e., no difference between sexes), we have included equivalence testing on torque‐related variables, to determine whether differences between sexes in such variables were so small as to not be practically meaningful. In all cases, equivalence tests yielded non‐significant results. Thus, while groups were not different (not significant null‐hypothesis testing), at the same time differences between sexes were not small enough for torque‐related metrics to be considered equivalent (not significant equivalence testing). The outcome of an equivalence test strongly depends on the width of the equivalence bounds [47]. The choice of a moderate relative effect size (0.6), justified by practically meaningful data [48] allowed rather liberal equivalence bounds (see Tables S4–S6). Despite this, relative torque‐related variables within voluntary and electrically evoked contractions were not equivalent between sexes.

According to previous research, pRTD (determined with a moving window) and pRTDf (determined from the RTD function) in voluntary contractions were achieved between 60–80 ms and 50–150 ms from contraction torque onset [39, 60]. Time to pRTD, but not to pRTDf, was shorter in males than females in all contraction types: ~13, 18, and 4 ms in explosive‐impulse, explosive‐hold, and electrically evoked contractions, respectively. Such differences may be driven by the higher type II/type I muscle fiber area ratio in males in the tibialis anterior muscle (~20% higher in males; [11]), the main contributor to ankle dorsiflexion with the joint angle used in the present study [61], as type II fibers exhibit a higher rate of tension development than the type I counterpart [33]. Despite the similar nature (i.e., maximum slope), the calculations of pRTD and pRTDf greatly differ: The former variable is based on a moving window of relatively narrow width and it is more transient than the latter variable, calculated as the slope of the tangent to the torque trace passing from its onset. This may explain the greater sensitivity of pRTD to differences in motor units twitch kinetics. Such hypothesis, however, needs to be verified experimentally.

### 4.2 Comparison Between Voluntary and Electrically Evoked Explosive Contractions

Comparisons between voluntary and electrically evoked contractions are often used (e.g., in studies of fatigue, post‐activation potentiation, or rapid torque production; [4, 62, 63, 64]) as trains of stimuli or tetani allow studying the response of the neuromuscular system under a deterministic neural input to muscle fibers. In the present research, the deterministic input was represented by trains of 50 pulses at 200 Hz to achieve the maximal possible evoked pRTD in the ankle dorsiflexors. Frequencies above 200 Hz were used for the same purpose when examining electrically evoked rapid thumb adduction (15 pulses at 250 Hz; [62]) or knee extension (octets at 300 Hz; [4, 63]).

For comparisons between males and females, electrically evoked contractions yielded the same findings of voluntary efforts: absence of sex differences in torque‐related variables when accounted for MVT, shorter time to pRTD but not time to pRTDf in males. Across sexes, time to pRTD and pRTDf were shorter (more than halved) in electrically evoked compared to voluntary contractions, and RTD and IMP were higher in the first 50 ms after torque onset (~1.5 times higher RTD and about twice the impulse) but lower in later time windows. Thus, electrically evoked contractions were characterized by different kinetics compared with voluntary contractions, shifting torque rise earlier after torque onset. These findings agree with those of de Ruiter et al. [63] and Baudry & Duchateau [62]. In the electrically evoked contractions in the present study, MUDR was comparable to the maximal observed from the first 2–3 pulses in explosive contractions in vivo (between 120 and 200 Hz; [39, 65]) or after intense current injection into motoneurones [66]. As a consequence of supramaximal stimulation, within the trains of stimuli motor units were all instantly recruited (rate of recruitment → ∞), with perfect synchronization (spike train correlation = 1). Thus, high discharge rate, instantaneous rate of recruitment, and perfect synchronization modified the kinetics of the rapid contractions, lowering pRTD but yielding a greater proportion of total impulse earlier in the contraction. These findings complement conclusions from earlier modeling studies, that numerically modified the rate of recruitment of motor units in explosive contractions and enhanced their synchronization by increasing common synaptic input to the motoneuron pool [67, 68]. Overall, findings indicate that higher motor units recruitment speed is favorable for rapid torque production, however, recruitment speeds above a certain threshold, with high level of motor units synchronization (in contractions where synchronization between motor units is already inevitably high; [69]) may not further improve pRTD, but may shift the kinetics of the explosive contraction, yielding greater impulse in the first 50 ms from onset. Within physiologically high rate of recruitment and synchronization of motor units, temporal dispersion of motor unit twitches may guarantee impulse and RTD late in the contraction, as shown by voluntary: evoked ratios lower than 1 in the window 0–50 ms from torque onset for RTD and 0–50 and 0–100 ms for IMP, but greater than 1 in the windows 0–100 and 0–150 ms for RTD and 0–150 ms for IMP (Table 3). Notably, observations from the present and earlier cited studies are mainly based on motor unit recruitment speed and synchronization induced by peripheral stimulation, and measured with EMG or modeled peripherally from the sarcolemma, and may not represent the actual recruitment speed and synchronization at the motoneuron level, which may differ because of differences in axonal and sarcolemmal conduction velocity between motor units.

### 4.3 Discharge Rate of Motor Units

The number of identified motor units per participant was lower in females (~4 ± 2) than in males (~6 ± 2). The lower motor units yield for females than for males is a common finding in most previous studies regarding high‐density EMG [17]. No differences between sexes were found for MUDR, at the beginning of both explosive‐impulse and explosive‐hold contractions, and at the plateau phase of explosive‐hold contractions. Values of MUDR in the present study are in line with those previously reported [32, 34, 59].

Previous research has reported differences in MUDR between sexes, but with the direction and magnitude depending on the muscle group examined, contraction intensity, and age [18]. In isometric ankle dorsiflexions, values of MUDR in the tibialis anterior were higher in females at relatively low submaximal intensities (20%–50% MVT; [15, 17, 70]), although similar MUDR between sexes has also been reported at 30% MVT [24]. At relatively higher submaximal intensities (50%–80% MVT), mean MUDR was comparable between sexes [15, 70], while it was higher in males than females in maximal efforts [14, 15]. Similar results are found for other muscles of the lower limb, for example, vastus lateralis in isometric knee extensions [19, 20, 23]. Across all contraction intensities, when strength‐matched with males, females report greater MUDR than males (from tibialis anterior; [15]).

At the plateau phase of explosive‐hold contractions, participants targeted 75% MVT. The absence of sex differences in mean MUDR of tibialis anterior found in the present study is in line with previous findings when targeting a similar submaximal contraction ankle dorsiflexion intensity (80% MVT; [15]).

In the present study, values of MUDR at the beginning of explosive efforts were not statistically different between males and females. Future research should investigate MUDR during explosive efforts in muscles other than the tibialis anterior where not only the percentage of muscle CSA occupied by type II fibers is greater in males than females [11], but also the type II/I fiber content ratio, such as typically shown from knee extensors [10, 23].

### 4.4 Comparison Between Voluntary Contraction Types

Values of MUDR at the onset of explosive efforts were significantly higher (~6 Hz) in explosive‐impulse than explosive‐hold contractions. To investigate further, we rerun the liner mixed‐effects model to compare contraction types (with data pooled between sexes) but excluding electrically evoked contractions. All torque‐related variable and RMS EMG were not significantly different between explosive‐impulse and explosive‐hold contractions (*p* > 0.07). As intent is a key predictor of explosive performance [71], the intent to contract “fast” in explosive‐impulse contractions as opposed to “fast and hard” or “fast and hold,” to keep a specified torque level in explosive‐hold contractions [72, 73] may manifest in a poorer explosive performance. While such difference in intent may have been enough to yield significantly lower MUDR in explosive‐hold contractions (~6 Hz), the effect may have not been practically relevant to affect either torque‐related variables or RMS EMG. Notably, even with a considerable increase in mean values of MUDR (~25 Hz) with a startle response, the maximum RTD (pRTDf) was only increased by ~15% [40]. Notwithstanding, future studies should evaluate both explosive‐impulse and explosive‐hold contractions.

### 4.5 Methodological Considerations

High‐density EMG is known to have a relatively low yield of motor unit numbers in females [17, 43]. To remedy this, we recruited a priori almost twice the number of females than males. Despite this, the known challenge of identifying motor units from high‐density EMG decomposition in ballistic contractions remains. Also, given that ballistic contractions imply maximal descending drive, the findings from this study may be skewed toward high‐threshold motor units. Additionally, differences in the recruitment rate and number of activated motor units between sexes may be a factor affecting explosive strength in the present study, not measured due to the limited amount of motor units identified per person. Sex differences in tendon stiffness may have also played a role in the comparison between sexes in the present study. Females typically present lower tendon stiffness and Young's modulus in the Achilles and patellar tendons, even when determined at comparable standardized torques [74]. Whether tendon mechanical properties in ankle dorsiflexor muscles are different between sexes remains to be determined. However, greater in‐series compliance slows the time course of force rise [75], which may pose an intrinsic limit to rapid torque production in females compared to males even in the case of comparable muscle properties. Findings from this study should be extended to other muscles and participants ages, as sex differences in motor unit behavior may be driven by the balance between type I and II fiber areas within the muscle, and may be differently affected by age [16, 76]. Future studies should ideally consider different phases of the menstrual cycle in females, as values of MUDR have been found to differ across menstrual phases [77]. The absence of a circular normal distribution based on the time after the last ovulation may not prevent testing females in a specific phase of the cycle, given individual variations in hormonal profiles [78]. Lastly, MVT was included as a predictor in the linear mixed‐effects models used. However, whether MVT should be treated as a confounder, mediator, or collider [79] in the comparisons between sexes remains to be determined. Matching males and females for MVT represents a valuable approach to conduct sex‐related comparisons.

## 5 Conclusions

No sex differences were found in rapid torque production, muscle activity, and motor units discharge rate during explosive isometric contractions of the ankle dorsiflexors. Relative torque‐related metrics were not equivalent between males and females for both voluntary and electrically evoked contraction types. Despite known differences between males and females in motor units firing rate in submaximal and maximal contractions of the ankle dorsiflexors, rapid torque production is comparable but not equivalent, and the control of the maximal rate of force production at the onset of an explosive task is not different between sexes.

## 6 Perspective

Explosive strength—the fundamental neuromuscular ability of producing the highest amount of force in the minimum time—is crucial for both athletic performance and clinical outcomes. When accounting for differences in maximal strength, males and females exhibit comparable capacities for rapid force production. This ability arises from the interplay of multiple elements along the neuromechanical continuum, from the motor cortex to muscle fibers. Within this continuum, female muscles typically exhibit a higher proportion of type I to type II fiber cross‐sectional area compared to males, resulting in slower contraction kinetics. Given this, we hypothesized that females would compensate for their slower muscle force kinetics with a higher motor unit discharge rate at the onset of explosive contractions. However, contrary to our expectations, our findings reveal no significant sex differences in motor units discharge rate during explosive ankle dorsiflexions. This suggests that despite inherent differences in muscle fiber composition and contraction kinetics, the neuromuscular control of rapid force production remains similar between sexes. Consequently, these findings indicate that training and conditioning for explosive strength may not require sex‐specific adaptations.

## Conflicts of Interest

The authors declare no conflicts of interest.

## Supporting information


Table S1.–S8.

## Data Availability

All data supporting the results presented in the manuscript are included in the manuscript figures. Data is available from the authors upon request.
