# Supplementary material for: No Differences in Motor Units Discharge Rate Between Females and Males in Explosive Ankle Dorsiflexions
Source: Scand J Med Sci Sports. 2025 May 11;35(5):e70065. doi: 10.1111/sms.70065 (PMC12066930; doi:10.1111/sms.70065)
Supplement: Supplementary file 1 — Table S1.–S8. [file SMS-35-e70065-s001.docx]

**No differences in motor units discharge rate between females and males in explosive ankle dorsiflexions.**

Anna Grootenhuis^1^, Fiona C. Hiereth^1^, Jakob Škarabot^2^, Marius Oßwald^3^, Alessandro Del Vecchio^3^, Markus Gruber^1^ and Luca Ruggiero^1^

^1^Human Performance Research Centre, Department of Sport Science, University of Konstanz, Konstanz, Germany.

^2^School of Sport, Exercise and Health Sciences, Loughborough University, Loughborough, United Kingdom.

^3^Neuromuscular Physiology and Neural Interfacing (N-squared) Laboratory, Department of Artificial Intelligence in Biomedical Engineering, Friedrich-Alexander-Universität, Erlangen, Germany.

**SUPPLEMENTARY MATERIAL**

**S1 – Results from the statistical analyses.**

*Maximal torque and M_max_ peak to peak amplitude*. Males had higher MVT and M_max_ peak-to-peak amplitude than females (MVT: *t* = 7.02, *P* < 0.01; M_max_ peak-to-peak amplitude: *t* = 3.14, *P* < 0.01). Outcomes for the statistical analyses of the peak torque of 10-Hz and 200-Hz trains are reported in Table S1. Since MVT was a significant predictor, only *t* and *P* values relative to the fixed factor of Sex.

Results from all other statistical analyses are reported in Tables S1, S2, and S3. In all linear mixed-effects models, when the fixed factors of either MVT or M_max_ peak-to-peak amplitude were present, these measures were significant predictors of the dependent variable. For this reason, only *t* and *P* values relative to the fixed factors of Sex and Contraction type are reported in the tables. Outputs from the equivalence tests for relative torque-related measures within contraction types are reported in Table S4, S5, and S6.

TABLE S1. Statistical outcomes for the linear mixed model (Dependent variable ~ 1 + Sex ⋅ MVT or Sex ⋅ M_max_ peak-to-peak amplitude) comparing torque-related variables and RMS EMG between sexes within the voluntary and electrically evoked contractions. Since no significant Sex ⋅ MVT or Sex ⋅ M_max_ peak-to-peak amplitude interactions were found, statistical regressions were re-run including fixed factors but removing the interaction from the model equations. Given that both MVT or M_max_ peak-to-peak amplitude were significant predictors of the dependent variable in all cases, only *t* and *P* values for the fixed factor Sex are reported.

| Variables | | *Explosive-impulse* | | *Explosive-hold* | | *10-Hz trains* | | *200-Hz trains* | |
| --- | --- | --- | --- | --- | --- | --- | --- | --- | --- |
|  |  | *t* | *P* | *t* | *P* | *t* | *P* | *t* | *P* |
| Peak torque |  |  |  |  |  | 0.56 | 0.57 | 1.46 | 0.16 |
| pRTD |  | 1.60 | 0.12 | 1.29 | 0.21 |  |  | 1.78 | 0.09 |
| pRTDf |  | 1.60 | 0.11 | 0.55 | 0.58 |  |  | 0.86 | 0.39 |
| RTD | 0-50 ms | 0.08 | 0.94 | 1.23 | 0.22 |  |  | 1.90 | 0.07 |
|  | 0-100 ms | 0.85 | 0.40 | 0.57 | 0.57 |  |  | 1.81 | 0.08 |
|  | 0-150 ms | 0.58 | 0.57 | 0.90 | 0.37 |  |  | 1.49 | 0.15 |
| IMP | 0-50 ms | 0.11 | 0.91 | 1.16 | 0.25 |  |  | 1.45 | 0.16 |
|  | 0-100 ms | 0.64 | 0.52 | 0.69 | 0.49 |  |  | 1.91 | 0.07 |
|  | 0-150 ms | 0.71 | 0.48 | 0.72 | 0.47 |  |  | 1.82 | 0.08 |
| RMS HDEMG | 0-50 ms | 0.66 | 0.51 | 1.16 | 0.25 |  |  |  |  |
|  | 0-100 ms | 1.06 | 0.29 | 0.85 | 0.39 |  |  |  |  |
|  | 0-150 ms | 1.34 | 0.19 | 1.00 | 0.32 |  |  |  |  |

pRTD, peak rate of torque development; pRTDf, peak rate of torque from the RTD function; IMP, torque impulse; RMS EMG, root-mean-square from the high-density electromyography signal.

TABLE S2. Statistical outcomes for the linear mixed model (Dependent variable ~ 1 + MVT + Contraction type + (1 | Participant); with M_max_ peak-to-peak amplitude instead of MVT as fixed factor for comparison of RMS EMG between explosive-impulse and explosive-hold contractions) comparing torque-related and RMS EMG variables between contraction types. Given the absence of sex-related differences when accounting for the variance explained by MVT or M_max_ peak-to-peak amplitude, data were pooled between sexes. Since MVT and M_max_ peak-to-peak amplitude were significant predictor of the dependent variable in all cases, only *t* and *P* values for the fixed factor of Contraction type are reported. Within the Contraction type category, explosive-impulse, explosive-hold, and electrically evoked contractions were identified as Type 1, 2, and 3, respectively.

| Variables | | *Type 1 vs. Type 2* | | *Type 1 vs. Type 3* | | *Type 2 vs. Type 3* | |
| --- | --- | --- | --- | --- | --- | --- | --- |
|  |  | *t* | *P* | *t* | *P* | *t* | *P* |
| pRTD |  | 1.78 | 0.07 | 3.71^**^ | <0.01 | 2.09^*^ | <0.05 |
| pRTDf |  | 1.78 | 0.08 | 0.49 | 0.62 | 0.16 | 0.87 |
| RTD | 0-50 ms | 1.65 | 0.10 | 6.00^**^ | <0.01 | 7.34^**^ | <0.01 |
|  | 0-100 ms | 1.47 | 0.15 | 5.02^**^ | <0.01 | 4.01^**^ | <0.01 |
|  | 0-150 ms | 0.57 | 0.57 | 10.6^**^ | <0.01 | 10.5^**^ | <0.01 |
| IMP | 0-50 ms | 1.52 | 0.13 | 10.28^**^ | <0.01 | 11.13^**^ | <0.01 |
|  | 0-100 ms | 1.64 | 0.10 | 2.27^*^ | <0.05 | 3.53^**^ | <0.01 |
|  | 0-150 ms | 1.40 | 0.16 | 3.25^**^ | <0.01 | 2.30^*^ | <0.05 |
| RMS EMG | 0-50 ms | 1.62 | 0.11 |  |  |  |  |
|  | 0-100 ms | 1.70 | 0.07 |  |  |  |  |
|  | 0-150 ms | 1.71 | 0.07 |  |  |  |  |

pRTD, peak rate of torque development; pRTDf, peak rate of torque from the RTD function; IMP, torque impulse; RMS EMG, root-mean-square from the high-density electromyography signal; Type 1, explosive-impulse contraction; Type 2, explosive-hold contraction; Type 3, electrically evoked contraction. ^*^*P* < 0.05; ^**^*P* < 0.01.

TABLE S3. Statistical outcomes for the linear mixed models comparing time to pRTD between contractions, MUDR at the beginning of explosive-impulse and explosive-hold contractions (Dependent variable ~ 1 + Sex ⋅ Contraction type + (1 | Participant)), and MUDR in the plateau phase of explosive-hold contractions between sexes (Dependent variable ~ 1 + Sex). Since no significant Sex ⋅ Contraction type interaction was present, only *t* and *P* values for the fixed factors Sex and Contraction type are reported. Within the Contraction type category, explosive-impulse, explosive-hold, and electrically evoked contractions were identified as Type 1, 2, and 3, respectively.

| Variables | | *Sex* | | *Contraction type* | | | | | |
| --- | --- | --- | --- | --- | --- | --- | --- | --- | --- |
|  |  |  |  | *Type 1 vs. Type 2* | | *Type 1 vs. Type 3* | | *Type 2 vs. Type 3* | |
|  |  | *t* | *P* | *t* | *P* | *t* | *P* | *t* | *P* |
| Time to pRTD |  | 3.58 | <0.01^**^ | 0.5 | 0.61 | 11.8^**^ | <0.01 | 10.9^**^ | <0.01 |
| Time to pRTDf |  | 0.97 | 0.33 | 1.12 | 0.28 | 11.2^**^ | <0.01 | 12.4^**^ | <0.01 |
| MUDR | onset | 1.19 | 0.23 | 2.14^*^ | <0.05 |  |  |  |  |
|  | plateau | 0.73 | 0.46 |  |  |  |  |  |  |

pRTD, peak rate of torque development; pRTDf, peak rate of torque from the RTD function; MUDR, motor units discharge rate; Type 1, explosive-impulse contraction; Type 2, explosive-hold contraction; Type 3, electrically evoked contraction. ^*^*P* < 0.05; ^**^*P* < 0.01.

TABLE S4. Statistical outcomes for the equivalence tests (two one-sided *t*-tests) comparing torque-related variables normalized to MVT between sexes in explosive-impulse contractions. Outcomes are reported for both lower and upper bounds.

| Variables | | *Equivalence bounds* | *95% CI Mean Difference* | | *P-value* | |
| --- | --- | --- | --- | --- | --- | --- |
|  |  |  | *Lower* | *Upper* | *Lower* | *Upper* |
| pRTD |  | ±0.842 | -0.645 | 1.330 | <0.05 | 0.16 |
| pRTDf |  | ±0.353 | -0.590 | 0.380 | 0.16 | <0.05 |
| RTD | 0-50 ms | ±0.546 | -0.746 | 0.534 | 0.09 | <0.05 |
|  | 0-100 ms | ±0.239 | -0.456 | 0.105 | 0.33 | <0.01 |
|  | 0-150 ms | ±0.401 | -0.650 | 0.290 | 0.18 | <0.05 |
| IMP | 0-50 ms | ±0.0005 | -0.0008 | 0.0004 | 0.17 | <0.05 |
|  | 0-100 ms | ±0.0022 | -0.0031 | 0.0021 | 0.10 | <0.05 |
|  | 0-150 ms | ±0.0039 | -0.0063 | 0.0029 | 0.17 | <0.05 |

CI, confidence intervals; pRTD, peak rate of torque development; IMP, torque impulse.

TABLE S5. Statistical outcomes for the equivalence tests (two one-sided *t*-tests) comparing torque-related variables normalized to MVT between sexes in explosive-hold contractions. Outcomes are reported for both lower and upper bounds.

| Variables | | *Equivalence bounds* | *95% CI Mean Difference* | | *P-value* | |
| --- | --- | --- | --- | --- | --- | --- |
|  |  |  | *Lower* | *Upper* | *Lower* | *Upper* |
| pRTD |  | ±0.548 | -0.916 | 0.368 | 0.21 | <0.01 |
| pRTDf |  | ±0.316 | -0.684 | 0.184 | 0.38 | <0.01 |
| RTD | 0-50 ms | ±0.503 | -1.006 | 0.174 | 0.39 | <0.01 |
|  | 0-100 ms | ±0.432 | -0.925 | 0.087 | 0.48 | <0.01 |
|  | 0-150 ms | ±0.271 | -0.691 | 0.056 | 0.74 | <0.01 |
| IMP | 0-50 ms | ±0.0004 | -0.0010 | 0.0010 | 0.49 | <0.01 |
|  | 0-100 ms | ±0.0023 | -0.0045 | 0.0009 | 0.36 | <0.01 |
|  | 0-150 ms | ±0.0043 | -0.0094 | 0.0007 | 0.51 | <0.01 |

CI, confidence intervals; pRTD, peak rate of torque development; IMP, torque impulse.

TABLE S6. Statistical outcomes for the equivalence tests (two one-sided *t*-tests) comparing torque-related variables normalized to MVT between sexes in electrically evoked contractions. Outcomes are reported for both lower and upper bounds.

| Variables | | *Equivalence bounds* | *95% CI Mean Difference* | | *P-value* | |
| --- | --- | --- | --- | --- | --- | --- |
|  |  |  | *Lower* | *Upper* | *Lower* | *Upper* |
| pRTD |  | ±1.029 | -1.134 | 1.693 | <0.05 | 0.16 |
| pRTDf |  | ±0.762 | -1.367 | 0.729 | 0.21 | <0.05 |
| RTD | 0-50 ms | ±0.707 | -0.975 | 0.969 | 0.09 | 0.08 |
|  | 0-100 ms | ±0.576 | -0.562 | 1.020 | <0.05 | 0.20 |
|  | 0-150 ms | ±0.451 | -0.352 | 0.888 | <0.05 | 0.29 |
| IMP | 0-50 ms | ±0.0009 | -0.0016 | 0.0009 | 0.21 | <0.05 |
|  | 0-100 ms | ±0.0032 | -0.0041 | 0.0046 | 0.07 | 0.10 |
|  | 0-150 ms | ±0.0062 | -0.0065 | 0.0165 | <0.05 | 0.17 |

CI, confidence intervals; pRTD, peak rate of torque development; IMP, torque impulse.

**S2 – Results from bipolar surface EMG analyses.**

*M_max_ peak to peak amplitude*. No differences were found between males and females for the peak-to-peak amplitude of the M_max_ measured from bipolar EMG (2.4 ± 0.7 vs. 2.3 ± 0.7 mV; *t* = 0.29, *P* = 0.77).

*Explosive contractions:* Muscle activity (RMS EMG) normalised to the peak to peak amplitude of the M_max_ (in A.U.) was 0.08 ± 0.03, 0.10 ± 0.02, and 0.11 ± 0.02 for males 0.07 ± 0.03, 0.11 ± 0.03, and 0.13 ± 0.03 for females in explosive-impulse contractions in the time windows 0-50, 0-100 and 0-150 ms from torque onset. In explosive-hold contractions, muscle electrical activity was 0.07 ± 0.03, 0.09 ± 0.03, and 0.11 ± 0.03 for males 0.07 ± 0.03, 0.11 ± 0.04, and 0.12 ± 0.04 for females within 0-50, 0-100 and 0-150 ms from torque onset. No differences between sexes were found (Table S7).

*Comparison between contraction types*. Normalised RMS EMG, pooled across sexes, was 0.08 ± 0.03, 0.11 ± 0.03, and 0.12 ± 0.03 for explosive-impulse contractions, and 0.07 ± 0.03, 0.10 ± 0.03, and 0.12 ± 0.04 for explosive-hold efforts, with no significant differences between contraction types (Table S8).

TABLE S7. Statistical outcomes for the linear mixed model (Dependent variable ~ 1 + Sex ⋅ M_max_ peak-to-peak amplitude) comparing RMS from surface bipolar EMG between sexes within the voluntary contractions. Since no significant Sex ⋅ M_max_ peak-to-peak amplitude interaction was found, the statistical regression was re-run including fixed factors but removing the interaction from the model equations. Given that M_max_ peak-to-peak amplitude was a significant predictor of the dependent variable in all cases, only *t* and *P* values for the fixed factor Sex are reported.

| Variables |  | *Explosive-impulse* | | *Explosive-hold* | |
| --- | --- | --- | --- | --- | --- |
|  |  | *t* | *P* | *t* | *P* |
| RMS EMG | 0-50 ms | 1.06 | 0.29 | 0.24 | 0.80 |
|  | 0-100 ms | 0.38 | 0.70 | 1.05 | 0.30 |
|  | 0-150 ms | 1.22 | 0.23 | 1.37 | 0.17 |

RMS EMG, root-mean-square from the high-density electromyography signal.

TABLE S8. Statistical outcomes for the linear mixed model (Dependent variable ~ 1 + M_max_ peak-to-peak amplitude + Contraction type + (1 | Participant)) comparing RMS from bipolar EMG between contraction types. Given the absence of sex-related differences when accounting for the variance explained by M_max_ peak-to-peak amplitude, data were pooled between sexes. Since M_max_ peak-to-peak amplitude was a significant predictor of the dependent variable, only *t* and *P* values for the fixed factor of Contraction type are reported. Within the Contraction type category, explosive-impulse and explosive-hold contractions were identified as Type 1 and 2, respectively.

| Variables |  | *Type 1 vs. Type 2* | |
| --- | --- | --- | --- |
|  |  | *t* | *P* |
| RMS EMG | 0-50 ms | 1.70 | 0.10 |
|  | 0-100 ms | 1.20 | 0.25 |
|  | 0-150 ms | 1.51 | 0.13 |

RMS EMG, root-mean-square from the high-density electromyography signal; Type 1, explosive-impulse contraction; Type 2, explosive-hold contraction.
